# A New Extract from Pomegranate (*Punica granatum* L.) By-Products as a Potential Oenological Tannin: Preliminary Characterization and Comparison with Existing Commercial Products

**DOI:** 10.3390/molecules25194460

**Published:** 2020-09-28

**Authors:** Valentina Canuti, Lorenzo Cecchi, Mohamad Khatib, Lorenzo Guerrini, Nadia Mulinacci, Bruno Zanoni

**Affiliations:** 1 DAGRI–Department of Agricultural, Food, Environmental, and Forestry Sciences and Technologies, University of Florence, via Donizetti, 6–50144 Firenze, Italy; Valentina.canuti@unifi.it (V.C.); lorenzo.guerrini@unifi.it (L.G.); Bruno.zanoni@unifi.it (B.Z.); 2 Department of Neurosciences, Psychology, Drug Research and Child Health (NEUROFARBA), Division of Pharmaceutical and Nutraceutical Sciences, University of Florence, via U. Schiff 6, 50019 Sesto Fiorentino, Italy; mohammadtttt@yahoo.com (M.K.); nadia.mulinacci@unifi.it (N.M.)

**Keywords:** polyphenols, ellagitannins, punicalagins, oenotannins, winemaking, by-products re-use, HPLC-DAD-MS, CIELab analysis, quality control, antioxidant capacity

## Abstract

Oenotannins are nowadays widely used as co-adjuvant in the winemaking process. The increasing cultivation and consumption of pomegranate (*Punica granatum* L.) leads to high amounts of by-products, mainly peels. Aim of this study was to propose a dried tannin-rich extract from pomegranate by-products as a new oenotannin. A decoction was prepared from mesocarp of the Wonderful pomegranate variety only using hot water as extracting solvent. The dried decoction was physical-chemically characterized and compared to 7 existing commercial oenotannins from different botanical origin (grape seed, oak, gallnut), according to UV-Vis Spectroscopy, Colorimetric analysis (CIELab), gelatin index, reactivity to proteins, HPLC-DAD, DPPH, and Total Polyphenols content by both Folin-Ciocalteu and the International Organization of Vine and Wine (OIV) methods. Data showed the lowest antiradical (AR) and total polyphenolic content (TPC) for the pomegranate tannin but also the highest AR/TPC ratio suggesting a good radical scavenging potential of this new tannin. Pomegranate tannin showed high reactivity with proteins, a unique chromatographic profile, and a low color impact. The new pomegranate tannin showed an interesting potential for oenological applications in particular for reducing the use of sulfites during vinification and for the white wine stabilization.

## 1. Introduction

The International Organization of Vine and Wine (OIV) authorized the use of oenological tannins to facilitate the clarification of wines and musts as long as they do not change the olfactory properties and the color of the wine [1]. Beyond this authorized use, nowadays oenotannins are commonly used in winemaking for other properties, classifiable in 5 main groups [2]: (i) impact on oxygen/metals: protection of wine from oxidation, anti-laccase activity, superoxide radicals scavenger, direct consumption of dissolved oxygen, iron-chelating ability and capability of preventing oxidative damages through Fenton-based reaction [3,4,5,6,7,8,9,10,11]; (ii) impact on color/pigments: improvement and stabilization of color in red wine, triggering for formation of new pigments and co-pigmentation effect [12,13,14,15]; (iii) protein interaction and preventing protein haze [16,17]; (iv) sensory/mouthfeel properties: capability of improving wine structure and mouthfeel (particularly bitterness and astringency) and of eliminating reduction odors [18,19]; (v) bacteriostatic effects [20].

Several types of oenological tannins are present in the market mainly differing in chemical structure, botanical origin, and/or preparation process. These include (i) hydrolyzable tannins, as gallotannins from oak galls, tara, myrabolan fruits, and nut galls, and ellagitannins from chestnut and oak; (ii) condensed tannins from grape seeds and skins, mimosa, quebracho, and acacia [2,11,12,15].

From the chemical point of view, condensed tannins (or proanthocyanidins) are polyhydroxyflavan-3-ol oligomers and polymers in which the flavanol subunits are linked through C-C bonds [21]. Hydrolyzable tannins are constituted of a glucose unit esterified by gallic acid moieties [22,23]. These, according to Okuda classification, are sub-classified as follow: gallotannins, characterized by several galloyl units linked each other by depside bonds (type-I hydrolyzable tannins) [24,25]; the intra- and/or intermolecular oxidative phenolic coupling to form C-C diaryl- and C-O diaryl- ether bonds between different galloyl residues gives rise to the formation of more than 1000 natural ellagitannins [26]. Ellagitannins are in turn divided into several sub-classes: type-II hydrolyzable tannins, characterized by the hexahydroxydiphenoyl (HHDP) group; type-III hydrolyzable tannins, characterized by dehydrohexahydroxydiphenoyl (DHHDP) group; type-IV hydrolyzable tannins, bearing for example the chebuloyl or elaeocarpusoyl groups; the *C*-glycosidic ellagitannins, in which the d-glucopyranose core is open and presents *C*-arylglucosidic bonds with galloyl-derived unit (as vescalagin and castalagin) [27]. The class of gallagyl esters presents the tetraphenyl ellagic acid-derived bislactone biester group (named gallagyl unit) as in pomegranate ellagitannins punicalin and punicalagins [27,28]. Complex tannins (or flavano-ellagitannins) are hydrolyzable tannins with a *C*-glucosidic ellagitannin moiety and a flavan-3-ol moiety. Finally, oligomeric ellagitannins can be formed through intermolecular C-O oxidative coupling between different groups in two monomers [22,23,29].

Depending on their chemical structure, the different oenotannins explain different mechanisms of action and different properties. Both condensed and hydrolyzable tannins have the capability to interact with proteins forming soluble and insoluble complexes, but with a different mechanism of action. Condensed tannins are well-recognized for their capability of reacting, directly or by means of acetaldehyde-mediated reactions, with anthocyanins in wines, forming stable polymeric pigments, thus resulting in enhancing color stability against oxidation during red wine aging [15]. Hydrolyzable tannins, and particularly ellagitannins, are able to regulate oxidation, to quickly react with dissolved oxygen, and to facilitate the hydroperoxidation of wine constituents inducing tannin/anthocyanin condensation, thus enhancing color stabilization and deepening the crimson color [12]. Ellagitannins have been reported as the most effective oenotannins in protecting wine against chemical oxidation [2,30]. The ellagitannin vescalagin reacts with the red-colored anthocyanin oenin (a grape pigment) to provide a purple-colored anthocyanin-ellagitannin pigment [31], suggesting the capability of ellagitannins to directly react with anthocyanins. Recently, hydrolyzable tannins also showed a high reactivity in co-pigmentation reactions with anthocyanins in wine [32].

Pomegranate tree (*Punica granatum* L., Punicaceae family) is native to central Asia, and thanks to its high adaptivity to a wide range of climates and soil conditions, it grows in many different geographical regions worldwide. Iran and India, but also America and the Mediterranean regions, are the main producers [33,34]. The demand for pomegranate keeps increasing year after year, also thanks to the increasing consumer awareness about the health benefits related to the pomegranate consumption, mainly in the form of juice [35,36,37,38,39,40]. Indeed, thanks to its high antioxidant capacity, pomegranate as a polyphenol-rich fruit is being commonly referred to as “superfruit” [28,41]. The edible part of the fruit consists of arils, mainly used for juice extraction, while the nonedible one, namely the peels (exocarp + mesocarp), accounts for approx. 40–50% of the total fresh fruit weight and to date it is usually discarded thus constituting the main by-product of juice extraction. In the recent years, the interest of the scientific community is focused not only on the characterization of the chemical constituents of the edible parts but also on the nonedible ones, in order to evaluate any possibility of re-using these parts [34,42]. If the arils juices are rich in anthocyanins [28,42], the fresh peel is constituted by ≈ 70% of water, high amounts of simple sugars, polysaccharides, and hydrolyzable tannins [28,43,44,45]. Overall, hydrolyzable tannins, mainly found in peels, is the predominant class of phenols into fruit, with very high amounts of ellagitannins of the gallagyl esters class, as punicalins and punicalagins [28,42]. These latter have been strongly correlated to the antioxidant capacity [28].

Among the hundreds of pomegranate varieties spread worldwide, Wonderful is one of the main cultivated throughout Europe [34]. In a recent study, it was highlighted the presence of high amounts of ellagitannins into the peels of this variety, mainly involving α- and β-punicalagins and 3 punicalagins’ derivatives [28]. The presence of this high content of ellagitannins suggests proposing the use of pomegranate peel extract as oenotannins aimed at improving the winemaking process.

The objective of this work was a preliminary evaluation of the suitability of a new pomegranate tannin extracted from mesocarp of the Wonderful variety for oenological applications. To this aim, a decoction from the fruit mesocarp was obtained and compared with several commercial oenotannins with different botanical origins.

## 2. Results and Discussion

**Figure 1:**
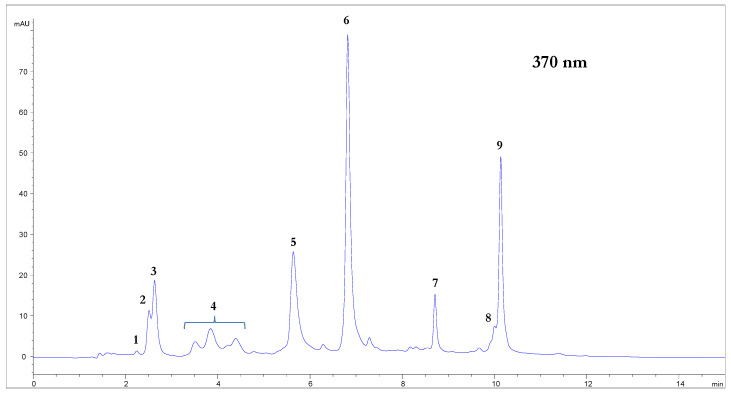
Chromatographic profiles at 370 nm of dried decoction with the main identified peaks: 1, gallic acid (identified in the chromatogram at 280 nm); 2, α-punicalin; 3, β-punicalin; 4, punicalagin derivatives; 5, α-punicalagin; 6, β-punicalagin; 7, ellagic acid hexoside; 8, ellagic acid pentoside; 9, ellagic acid.

The decoction obtained from the dried mesocarp of pomegranate fruit of the Wonderful variety was preliminarily analyzed in order to propose it for different applications. The proximate composition of the dried decoction was as follows: proteins 2.3%, total sugar 45.0%, total dietary fiber (measured with the AOAC 991.43 method) 9.7% (soluble 6.7%, insoluble 3.0%), while fats were not present in detectable amount, as expected. The content of ellagitannin was approximately 15%, almost completely represented by α + β punicalagins, ellagic acid, and α + β punicalins, as highlighted by Figure 1, showing the chromatographic profile at 370 nm obtained through analysis of the pomegranate dried extract by HPLC coupled Diode Array Detector and Mass Spectrometer Detector (HPLC-DAD-MS) (Figure 1).

A first comparison of the crude pomegranate tannin (TP) with the 7 commercial oenotannins (all listed in Table 1) of different botanical origin was based on their antioxidant and antiradical activities. Results of total phenolic content by Folin-Ciocalteu assay (TPC), total polyphenol index (TPI), and antiradical activity (AA) evaluated with 2,2-diphenyl-1-picrylhydrazyl (DPPH) are reported in Table 2, together with sample name, given code, and type of tannins. The AA/TPC ratio was also calculated in order to evaluate the antiradical activity on the basis of the total phenolic content.

The total phenolic content of TP was the lowest (273 mg/g), with the other tannins showed an average of 640 mg/g and the TNG sample as the one with the highest value (820 mg/g). The TPI reflects the same trend of TPC, with the TP that showed the lowest value (7.62) and TNG the highest one (26.64). The AA% shows the antiradicalic activity of the different oenological tannins according to the botanical origin: before measuring AA% of the tannins, we also measured the AA% of the model wine solution with no addition of any tannin, obtaining 2% of antiradical activity, this datum indicating that data in Table 2 (all higher than 68%) are due to tannins. The TNG showed the highest antiradical activity (93.7%), followed by the ellagic tannins, which showed an average value of 90.1%; finally, the TGS (79.5%) and the TP (68.2%) showed the lowest values. Despite this evidences, TP showed the highest AA/TPC ratio value of 0.25, indicating a higher reactivity of the pomegranate polyphenols against radicals in comparison to those of the other commercial tannins; on the opposite, the TNG, showing the highest TPC, is characterized by the lowest AA/TPC ratio. These evidences point out the possible use of these tannins during several phases of white, rosé, and red wine production, such as pre-fermentation, fermentation, aging, and so on. The wide variability of total phenols according to the botanical origin, and the highest TPC and AA activity showed by the TNG, are in agreement with previous literature [2,5,11].

**Figure 2:**
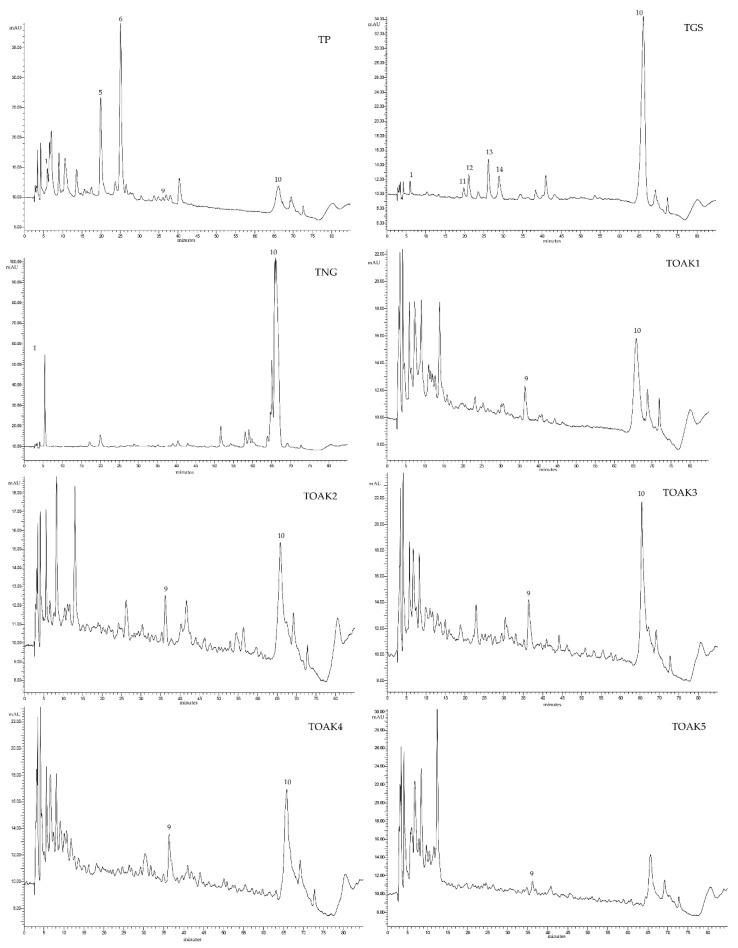
Chromatographic profiles at 280 nm of tannins in model wine solutions with the main peaks identified: 1, gallic acid; 5, α-punicalagin; 6, β-punicalagin; 9, ellagic acid; 10, polymeric tannins; 11, procyanidin B1; 12, (+)-catechin; 13, (−)-epicatechin; 14, procyanidin B2. TP, Pomegranate tannin; TGS, grape seed tannin; TNG, Nut gall tannin; TOAK1–5, Oak tannin 1–5.

All the 7 commercial tannins and the pomegranate tannin were also analyzed by RP-HPLC-DAD in order to compare the different phenolic composition. The used chromatographic method provides chromatograms that allow evidencing the monomeric and oligomeric phenols and the polymeric tannins (Figure 2, peak at 65 min), a method already used for analyzing oenotannins [12] and pigmented polymers in red wines [46]. Figure 2 shows the chromatograms registered at 280 nm of the eight different tannins. The different botanical origins can be immediately argued by observing the chromatograms: the ellagitannins showed very similar profiles among each other, with a lower content of polymeric tannins when compared to TNG. In fact, the polymeric tannins in TNG showed a chromatographic peak (peak n° 10) approximately 9 times greater compared to the tannins extracted from oak. The profile of the TGS was different from all the other ones and characterized by the presence of (+)-catechin, (−)-epicatechin, and procyanidins B1 and B2, very typical for a condensed tannin derived from grape, and by a moderate content in polymeric tannins. These findings are in agreement with previous literature [12]. As concerns the pomegranate tannin (TP), a very particular profile was evidenced as already described above: TP was composed by a lower content of polymeric tannins compared to all the others commercial products, very similar to the ellagic tannins, but higher in ellagitannins of the gallagyl type, characterized by a lower molecular weight [28].

**Figure 3:**
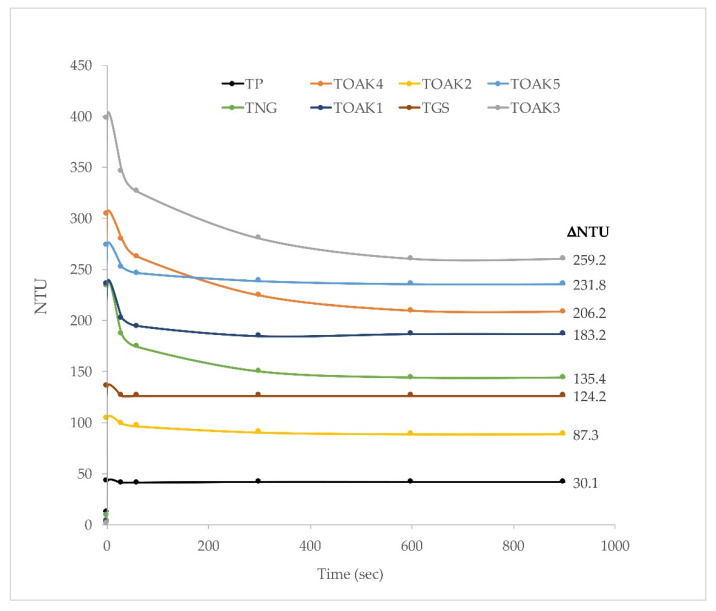
Reaction kinetics of the analyzed tannins dissolved in the model wine solution with gelatin from bovine serum albumin, and ΔNTU (nephelometric turbidity unit). Pomegranate tannin; TGS, grape seed tannin; TNG, Nut gall tannin; TOAK1, TOAK2, TOAK3, TOAK4, TOAK5, Oak tannin 1–5, respectively.

**Figure 4:**
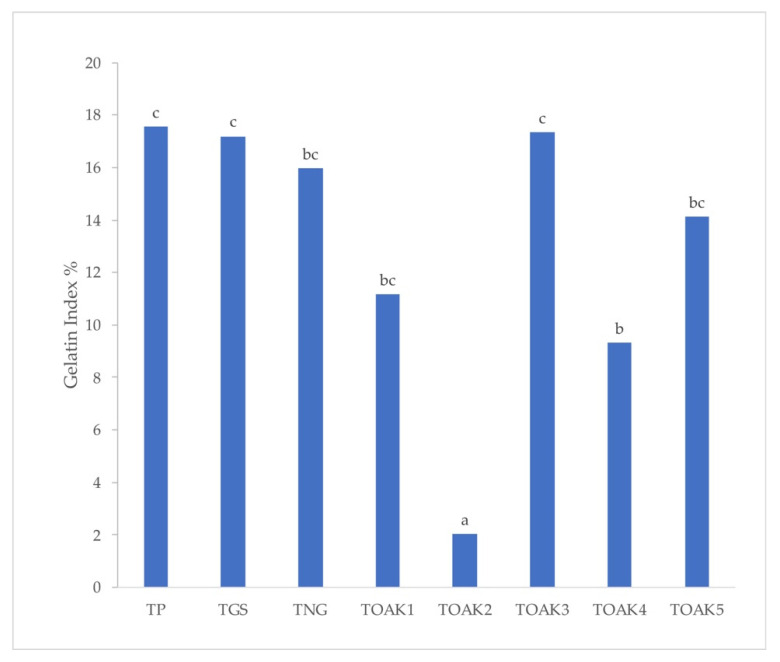
Gelatin index for the analyzed tannins dissolved in the model wine solution. Different letters indicate significant difference at *p*-value < 0.05. TP, Pomegranate tannin; TGS, grape seed tannin; TNG, Nut gall tannin; TOAK1–5, Oak tannin 1–5, respectively.

The above different composition of tannins is in agreement with the literature [47] and is able to cause different properties linked to a different reactivity of the molecules. For example, the capability of tannins to precipitate proteins depending on the protein to tannin ratio and on the type of tannins is well-known [15]. For this reasons, the pomegranate tannin was compared to the 7 commercial products for its capability in precipitating bovine serum albumin (BSA): the higher the capability in precipitating BSA, the higher the protein stabilization capability of the tannin when added to wine and the greater the astringency [47]. The different reactivity with proteins was evaluated with two different approaches: (i) the typical gelatin index, which is the more used despite several other approaches have been proposed over time [47,48]; (ii) the measurement of the evolution of turbidity after addition of BSA to the tannin solution for the evaluation of the capability of both helping in protein fining and preventing protein haze [16]. The evolution of turbidity is shown by the curves in Figure 3, which also reports the ΔNTU (Nephelometric Turbidity Unit), calculated as the difference between turbidity before protein addition to the tannin solutions and turbidity after stabilization (i.e., approximately 900 s). It is interesting to highlight the wide variability in the observed ΔNTU values: overall, the oak tannins are the more reactive ones with ΔNTU in the range 259.2–183.2 (with the exception of TOAK2, with ΔNTU of 87.3). The TNG is quite lower than the oak tannins and with a value (135.4) similar to the TGS (124.2). Finally, the TP extract was the less reactive one with a ΔNTU of 30.1; the result about TP has to be related to its not so high phenolic concentration, probably due to the non-purified character of TP extract. Despite this lower ΔNTU of TP, Figure 4 shows that the gelatin index of TP was one of the highest, together with TGS and TOAK3, indicating a high reactivity of TP polyphenols and a consequent potentiality as a suitable oenotannin after optimizing its purification. This high reactivity, likely due to the lower molecular weight of TP polyphenols than those of the other commercial tannins, will make it necessary to evaluate the impact of TP in astringency perception after addition to wine [48].

**Figure 5:**
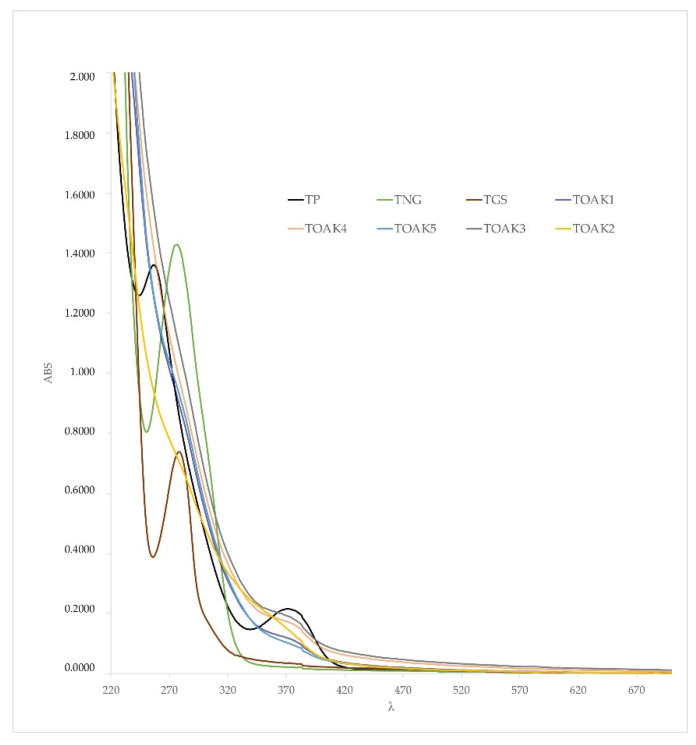
UV-Vis spectra of the analyzed tannins dissolved in the model wine solution. Pomegranate tannin; TGS, grape seed tannin; TNG, Nut gall tannin; TOAK1, TOAK2, TOAK3, TOAK4, TOAK5, Oak tannin 1–5, respectively.

In the next step of the study, we performed the color analysis in order to complete the characterization of the nature of the 8 different tannins. In fact, the UV-Vis spectroscopy is currently widely used to analyze oenotannins in order to differentiate them according to the botanical origin [49], while the CIEL*a*b* analysis is used to describe the color of the analyzed powder since the addition of tannins to wine might affect the color, which is not permitted by regulation [1,50]. Figure 5 reports the UV-Vis spectra of the 8 analyzed tannins: the TP showed minimum absorbances (λ_min_) at 243 nm and 339 nm and maximum absorbances (λ_max_) at 254 nm and 372 nm, with the latter being very close to the typical λ_max_ of punicalagins [28], in agreement with the polyphenolic profile above discussed. This profile is quite different from all the other 7 commercial tannins and also to other tannins reported in the literature [49], confirming that if this new tannin will be commercialized after purifaction it will be a diverse and easily recognizable oenotannin with respect to the existing commercial ones. Interestingly, all samples showed a very low absorbance starting from 420 nm, thus not affecting the color properties of wines in the visible region. As concern TP, despite the λ_max_ at 372 nm, it showed absorbance at 420 nm even lower than all the other tannins with the exception of TNG. Even considering a possible three-time increase of polyphenols concentration in TP (Table 2) after optimizing purification, and assuming a proportional increase of absorbance, the absorbance at 420 nm would remain lower than some of the analyzed commercial tannins.

**Figure 6:**
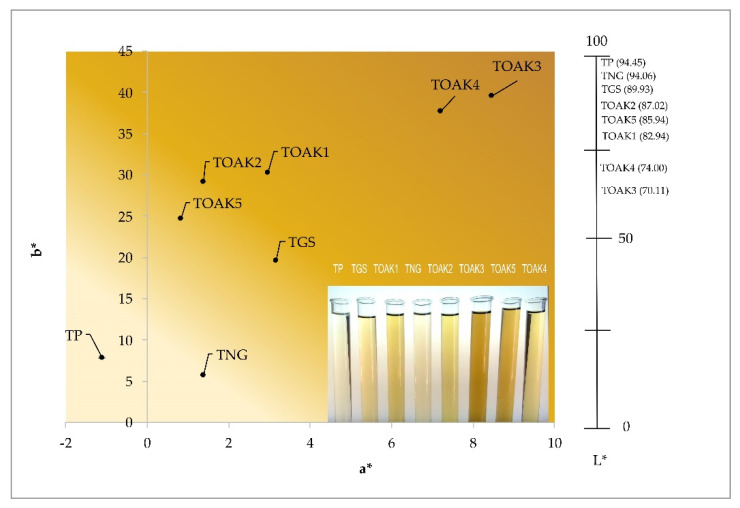
CIEL*a*b* coordinates of the analyzed tannins dissolved in the model wine solution. The 2D graph in the left reports the a* and b* coordinates, while the 1D axe in the right part reports the L* coordinate. An image of the analyzed tannin solutions is also shown in the bottom-right part of the 2D diagram. TP, Pomegranate tannin; TGS, grape seed tannin; TNG, Nut gall tannin; TOAK1–5, Oak tannin 1–5, respectively.

The CIEL*a*b* analysis has been carried out in order to better describe the color contribution of TP with respect to the other tannins. In fact, oenological tannins are well-recognized to be characterized by powders with quite different colors, for example ranging from pale-yellow to reddish-brown, which can differently affect the wines’ characteristics [51]. As shown in Figure 6, the TP is again quite differentiable from all the other tannins, with the more similar one being the TNG, in agreement with the above described UV-Vis spectra. In fact, the TP and TNG showed similar L* and b* coordinates, differing in a significant manner only for the a* coordinate, and resulting the less intense in color, as evidenced by the image of the eight solutions in Figure 6. This characteristic is very important since they would only slightly affect the wine color when added during production, in particular of white wines and spirits (i.e., grappa or brandy).

## 3. Materials and Methods

### 3.1. Chemicals

The Milli-Q-system (Millipore SA, Molsheim, France) was used to produce ultrapure water. Acetonitrile of both HPLC and HPLC-MS grades were purchased from Panreac (Barcelona, Spain). Formic acid and ethanol of analytical reagent grade were from Sigma-Aldrich (Steinheim, Germany). (+)-Catechin, (−)-epicatechin, procyanidin B1 and B2 were high in purity and purchased from Extrasynthèse (Genay, France). l-tartaric acid 99.5%, sodium carbonate ≥95%, Bovine Serum Albumin (BSA, ≥98%), gallic acid monohydrate ≥98%, α+β punicalagin ≥90%, ellagic acid ≥95%, 1,1-diphenyl-2-picrylhydrazyl radical (DPPH), and Folin-Ciocalteu reagent were purchased from Sigma-Aldrich (St. Louis, MO, USA). Polyvinylpolypyrrolidone (PVPP) was purchased from Enolife (Montemesola, Taranto, Italy). Seven commercial tannins were used in this study in addition to the new tannin from pomegranate (Table 1): one of the proanthocyanidin type (procyanidins/prodelphinidins from grape seed), and 6 hydrolyzable tannins among which one gallotannin from nut gall and 5 ellagitannins from oak.

### 3.2. Preparation, Characterization, and Preliminarily Purification of the Decoction from Pomegranate Mesocarp (Wonderful Variety)

Pomegranate fruit (Wonderful variety) were cultivated in Apulia region (Italy) and harvested in 2015. Mesocarp was manually separated from the other parts of the fruit (total 18 kg). The mesocarp was constituted by 80% of moisture and was used to prepare a decoction [38]: briefly, mesocarp was boiled in water for 60 min using an extractive ratio mesocarp/water of 1:40 *w/v*. The obtained mixture was cooled at room temperature, centrifuged for 2 min at 5000 rpm, and filtered; the solution was freeze-dried, thus obtaining a dried product (TP, Table 1) easily storable and usable in the powder form. The final yield of the extract was 75% on the dried mesocarp basis.

The proximate composition of the dried decoction was evaluated according to the following analysis: the Kjeldahl method was applied for determination of the protein content (PC = N × 6.25, where N is the total nitrogen and PC is the protein content in g/100 g); the content of fat was gravimetrically determined after Soxhlet extraction according to protocol ISS n° 1996/34; the soluble and insoluble dietary fiber was finally determined according to the method AOAC n° 991.43.

### 3.3. Preparation of Solution of the 8 Analyzed Tannins in Model Wine Solution

All tannins (the 7 commercial ones and the TP, Table 1) were dissolved in model wine solution as follow: 1 g of each tannin was dissolved in 1 L of the model wine solution constituted by ethanol (12% *v/v*) and 4 g/L of l-tartaric acid in water, with pH adjusted to 3.5 with NaOH.

### 3.4. Evaluation of Total Phenolic Content

#### 3.4.1. Folin-Ciocalteu Assay

The Folin-Ciocalteu method [52], slightly modified [53], was used for determining the total phenolic content. Deionized water (0.5 mL) and the Folin-Ciocalteu reagent (125 µL) were added in a 15 mL plastic flask together with 125 µL of the diluted sample extract. After 6 min, 1.25 mL of 7% aqueous Na_2_CO_3_ solution was added, and the final volume was brought to 3 mL with water and the mixture was left for 90 min. Absorption was measured at 760 nm against water as a blank. A calibration curve built with gallic acid (range 20 to 500 µg mL^−1^, R^2^ = 0.9969) was used for evaluating the amount of total phenolic compounds, which was expressed as gallic acid equivalents (GAE, mg gallic acid/g sample).

#### 3.4.2. Total Polyphenols Index

The total polyphenol index (TPI) was determined by measuring the 280 nm absorbance of a 1:100 dilution of tannin solutions with a spectrophotometer, using a 10 mm quartz cuvette and multiplying the absorbance value by 100 [54].

### 3.5. Evaluation of Antiradical Activity by DPPH

The DPPH• (1,1-diphenyl-2-picrylhydrazyl radical) assay was used for evaluating the free radical scavenging activity according to the procedure previously reported [55] and slightly modified. DPPH solution was prepared by dissolving 4 mg of solid material in 100 mL of ethanol; the obtained solution was kept overnight at 4 °C without light exposure, then it was stored at −20 °C and thawed at room temperature before use. One milliliter of sample solution (tannin in model wine) was mixed to 1 mL of an ethanolic solution of DPPH• (0.004 g/100 mL) and the mixture kept at room temperature. Absorption was measured at 517 nm immediately and after 20 min using a Lambda 25 spectrophotometer (PerkinElmer, Waltham, MA, USA) versus an ethanol:H_2_O 50:50 solution as a blank. The same procedure was also performed adding 1 mL of model wine instead of sample solution to compare the antiradical activity of tannins with that of the model wine itself. The absorption of the DPPH• solution was checked daily. The antiradical activity was expressed as percentage of antiradical activity, using the following formula:

(1) $$AA\left(\%\right)=\;\frac{A_{t0}-A_{t20}}{A_{t0}}\;\times 100$$

where AA(%) is the antiradical activity, At_0_ is the absorbance at time 0, and At_20_ is the absorbance after 20 min.

### 3.6. UV-Vis Spectroscopy

UV–Vis spectra of tannins were acquired in the range 200–700 nm using a 1 mm quartz cuvette (spectrophotometer Lambda 35 UV⁄Vis; PerkinElmer).

### 3.7. HPLC and HPLC-MS Analysis

Oenotannin preparations were analyzed by HPLC by a previous developed method [46]. HPLC analysis was carried out on a 200 LC system equipped with autosampler and diode-array detector (PerkinElmer). Prior to injection, oenotannin extracts solutions were centrifuged (13,148× *g*) and filtered at 0.22 μm. Injection volume was 20 μL, flow rate was 1 mL/min with the following gradient of solvent A (aqueous 1.5% (*v/v*) H_3_PO_4_) and solvent B (20% (*v/v*) solvent A in CH_3_CN): from 8 to 27% solvent B in the first 55 min, held isocratic at 27% from 55 to 59 min, increased from 27% to 70% from 59 to 64 min, held at 70% from 64 to 69 min, and reduced to 8% from 70 to 76 min. Chromatograms were acquired at 280 nm, recorded, and processed using Total Chrome Navigator software (PerkinElmer). LC-MS analysis was also performed for analyzing the TP, following the method recently reported [56]: briefly, an HP 1260 MSD mass spectrometer provided with both DAD and MSD detectors, and with an API/electrospray interface (Agilent Technologies, Palo Alto, CA, USA), was used. Compounds were separated in a Kinetex 100 EC-C18 (30 × 3 mm, 2.6 µm, Agilent, USA) column. Solvent A was acetonitrile and solvent B was H_2_O acidified by HCOOH (3%, *v/v*). The elution was carried out as follows: 0–8 min, solvent A varied 5–25%; 8–18 min, stayed at 25%; 18–20 min varied 25–95%; 20–26 min stayed at 95%. Total analysis time, 28 min; equilibration time, 10 min; flow rate 0.4 mL/min. Injection volume was 2 µL. Chromatograms were recorded at 280, 370, 380, and 520 nm. ESI parameters: nitrogen flow rate 10.5 L/min, drying gas temperature 350 °C; nebulizer pressure, 1811 Torr; capillary voltage, 3500 V. Acquisition was performed in full spectrum scan (range 100–2000 Th) in negative ion mode with fragmentor voltage was set at 70 V or 300 V.

### 3.8. CIEL*a*b* Coordinates

CIE (Commission Internationale de l’Eclairage) L*, a* and b* color coordinates were measured [57,58]. Visible spectra were recorded at 400–700 nm transmittance using a spectrophotometer Lambda 35 UV⁄Vis (PerkinElmer) equipped with the RSA-PE-20 Integrating Sphere accessory assembly (Labsphere, North Sutton, NH, USA). UV WinLab Software was used to record the spectra (version 2.85.04, PerkinElmer Inc.) and CIE L*a*b* color coordinates were calculated for the CIE illuminant D65 and 10° standard observed conditions, using Color software (version 3.00, 2001, PerkinElmer Inc.). Samples transmittance was measured using a 1 mm quartz cuvette.

### 3.9. Tannin Analysis by the OIV Method

The TP was also analyzed according to the official OIV methods [1]. Total solids (TS), soluble solids (SS), and non-phenolic solids (NPS) were determined using an SPE column with polyvinylpolypyrrolidone (PVPP) according to the official method. A blank measurement (BK) was also taken by doing the same as for the NPS. The tannin richness (%tannins) was estimated using the following equation:

%Tannins = (SS − NPS − BK)/TS × 100(2)

### 3.10. Gelatin Index and Turbidity

All the oenological tannins were analyzed using the gelatin precipitation method [59]. Briefly, 4 mL of oenological tannin solutions were placed in two centrifuge tubes. Tube A (sample) received an addition of 0.4 mL of aqueous BSA solution (7% *w/v*). Tube B (control) was prepared similarly, but the added BSA solution was replaced with water. After 24 h at room temperature, the two tubes were centrifuged, and the supernatants were diluted 1:100 with water and read at 280 nm in a 1 cm quartz cuvette, obtaining the absorbance values (A_0_ for tube B diluted solution, A for tube A diluted solution). The gelatin index was calculated according to the following formula:

Gelatin Index = ((A_0_ − A)/A_0_) × 100 (3)

This index gives information concerning the reactivity of the tannin: the higher the value, the higher the reactivity of the extract towards proteins.

The turbidity of Tube A was also measured before and after the addition of BSA solution and monitored for approximately 20 min, until turbidity stabilization, using a HACH2100N turbidimeter.

### 3.11. Statistical Analysis

All analyses were carried out in triplicate and the results were expressed as mean values. One-way ANOVA was applied to verify the existence of significant differences between samples; when the presence of differences was confirmed, Fisher’s LSD test was applied to differentiate between mean values.

## 4. Conclusions

In this work, an extract from pomegranate by-products obtained by a green decoction process was characterized and compared to a series of oenotannins with different botanical origins in order to explain its potential use as oenotannin.

The experimental data indicated that the raw pomegranate extract is a potential new oenological tannin with a different tannin composition compared to those commercially available so far, thus enlarging the variability of products available on the market. In fact, it is characterized by the presence of a peculiar class of ellagic tannins, namely those bearing the gallagyl group such as punicalins, punicalagins, and their derivatives. A high antiradical activity (almost 70%) and a color profile suitable for winemaking have been pointed out.

The raw extract should be purified in order to increase the polyphenols concentration for meeting the indication of the OIV. In fact, quantification of tannins by the OIV method showed that the total polyphenols content of the TP is quite low (approximately 15%), in agreement with the total phenolic content measured with the Folin-Ciocalteu method (273 mg/g). The measured composition of the raw extract indicated that about half of its weight is constituted by sugars, thus removing them may allow for a strong increase of tannins concentration. In the next steps of the work, the extraction and purification processes of the pomegranate tannin will be optimized in order to obtain a product richer in ellagitannins and free from impurity.

The use of oenotannins in winemaking has received renewed attention, and the winemaking process itself is daily facing against the use of synthetic antioxidant as sulfites. In this context, this study pointed out the possibility of recovering added value from a by-product of pomegranate consumption giving to the enology a new possibility. The new extract would be useful in several steps of winemaking process for allowing improving the protein stability of white and rosé wines, enhancing the color stability, and reducing the use of sulfites thanks to its capability of protection from oxidation. The addition of the tannin will also result in a higher content of polyphenols, commonly associated with wines with a higher market value. Its use in the winemaking process has to be carefully studied in order to propose it for the list of admitted oenotannins.

## Figures and Tables

**Table 1:** List of the analyzed tannins. For each tannin, the given code, the botanical origin, and the type of tannin are also reported.

Tannin Name	Code	Botanical Origin	Type of Tannin
Pomegranate tannin	TP	Pomegranate	Hydrolyzable (Ellagic)
Grape seed tannin	TGS	Grape seed	Condensed (procyanidin)
Nut gall tannin	TNG	Nut gall	Gallotannin
Oak tannin 1	TOAK1	Oak	Hydrolyzable (Ellagic)
Oak tannin 2	TOAK2	Oak	Hydrolyzable (Ellagic)
Oak tannin 3	TOAK3	Oak	Hydrolyzable (Ellagic)
Oak tannin 4	TOAK4	Oak	Hydrolyzable (Ellagic)
Oak tannin 5	TOAK5	Oak	Hydrolyzable (Ellagic)

**Table 2:** Antioxidant and antiradical activity of the analyzed tannins dissolved in the model wine solution. TPC: total phenolic content; TPI, total polyphenolic index; AA, antiradical activity evaluated using 2,2-diphenyl-1-picrylhydrazyl. Data are expressed as mean of three determinations; in each column, different letters (a–f) indicate statistically significant differences. *F*-values, *p*-values, and standard errors of means are also reported.

Sample Code	TPC (mg gallic acid/g)	TPI (Abs_280nm_)	AA (%)	AA/TPC
TP	273 a	7.62 a	68.2 a	0.25
TGS	601 c	15.38 c	79.5 b	0.13
TNG	820 e	26.64 h	93.7 e	0.11
TOAK1	608 cd	18.33 e	92.6 de	0.15
TOAK2	482 b	14.26 b	83.1 c	0.17
TOAK3	693 d	21.45 g	91.5 de	0.13
TOAK4	690 d	18.70 f	90.6 d	0.13
TOAK5	585 c	18.00 d	92.5 de	0.16
*F*-value	126.06	31124	97.28	
*p*-value	<0.0001	<0.0001	<0.0001	
Standard error	14.52	0.0002	1.17	
